# Prevalence of post-traumatic stress disorder among Palestinian children and adolescents exposed to political violence: A systematic review and meta-analysis

**DOI:** 10.1371/journal.pone.0256426

**Published:** 2021-08-26

**Authors:** Nisreen Agbaria, Stephanie Petzold, Andreas Deckert, Nicholas Henschke, Guido Veronese, Peter Dambach, Thomas Jaenisch, Olaf Horstick, Volker Winkler

**Affiliations:** 1 Institute of Global Health, Heidelberg Research to Practice Group, Heidelberg University, Heidelberg, Germany; 2 Institute for Musculoskeletal Health, School of Public Health, The University of Sydney, Sydney, Australia; 3 Department of Human Sciences & Education, University of Milano-Bicocca, Milan, Italy; 4 Section Clinical Tropical Medicine, Heidelberg University Hospital, Heidelberg, Germany; 5 Centre for Global Health, Colorado School of Public Health, Aurora, Colorado, United States of America; University of California, San Francisco, UNITED STATES

## Abstract

**Objective:**

We undertook a systematic review of the literature to explore the prevalence of post-traumatic stress disorder (PTSD) in Palestinian children and adolescents exposed to political violence. This is the first systematic review and meta-analysis of the prevalence of PTSD in this population.

**Methods:**

PubMed, Embase, PsycInfo, Google Scholar and Cochrane library were searched until June 2020. To estimate the prevalence of PTSD, sub-group and meta-analysis were conducted.

**Results:**

The search resulted in 2786 studies, of which 28 articles representing 32 samples with a total of 15,121 participants from Gaza Strip and West Bank met either the DSM-4 or DSM-5 criteria and were included. The pooled prevalence of PTSD was 36% (95% CI 30–41%; *I*^*2*^ 98.6%) and ranged from 6% to 70%. Sub-group analysis showed that the PTSD prevalence did not differ according to region (West Bank, Gaza Strip) and tended to decrease after including only studies using a representative sample (*p*<0.001), and among those with low risk of bias (*p*<0.001). Visual inspection of the included studies revealed significant discrepancies in study design and assessment measures.

**Conclusion:**

We identified high prevalence of PTSD among Palestinian children and adolescents exposed to political violence. However, the pooled results should be interpreted with caution, due to the high heterogeneity and risk of bias in the included studies. These limitations also reflect the challenge in conceptualizing and measuring PTSD in the Palestinian context with a background of continuous and cumulative trauma. Understanding the contextual factors and developing locally adapted survey measures are of relevance to future research, public health planning, and the provision of mental healthcare in Palestine.

## Introduction

Long-term exposure to political violence (EPV), such as the case of Israeli-Palestinian conflict, has led to profound physical and psychological distress to civilians [1], and results in the disruption of their social and economic structures [2]. Together and separately, these circumstances harm the mental health and development of children and youth [3]. The World Health Organization (WHO) recognizes political violence (PV) as major threat to public health, which includes acts of a physical, psychological, or sexual nature to achieve political goals [4]. In addition to its direct effects on children and adolescents, PV also affects collective well-being in various contexts of the children’s lives, such as the family, school, peer networks, and the entire environment in which they are raised [5]. Research suggests that children exposed to war-related trauma manifest mental disorders more frequently compared with children in the general population [6]. Moreover, adolescents exposed to PV have been found to be at risk for psychological distress and post-traumatic stress reactions [7].

Children and adolescents exposed to PV can experience a wide array of psychological conditions, including aggressive behaviors, depression, anxiety, learning difficulties and post-traumatic stress disorder (PTSD) [8]. PTSD remains the most commonly assessed and present psychological sequela [9–11]. PTSD is characterized by intrusions, avoidance of reminders of the event, and persistent symptoms of hypervigilance and arousal. Children may also exhibit physical symptoms such as stomach-aches and headaches [12]. A meta-analysis from 2009 of studies on 81,866 adults exposed to mass conflicts and displacement reported a PTSD prevalence of 30.6% [13]. Another meta-analysis reported the prevalence of PTSD to be about 36% among 34 samples of children and adolescents exposed trauma [14]. However, rates of PTSD vary widely depending on several factors, including participants’ age, the time elapsed since the traumatic event, and the version of the DSM diagnostic criteria used [15], as well as the sample characteristics, informant source, and the type of instrument used to assess PTSD [16].

The pervasive and long-standing Palestinian-Israeli conflict has exposed children and adolescents on both sides to PV [2]. The occupied Palestinian territories (oPt) (West Bank, East Jerusalem, and the Gaza Strip) were occupied by Israel in 1967, and has been the place of periods of significance escalations in violence since, including two major Palestinian uprisings (also called *Intifadas*) against the occupation, first from 1987 to 1993 and then from 2000 to 2005. Since 2007, the Gaza Strip (GS) has witnessed recurring wars, which took place in 2008, 2012, and 2014. Aside from these major events, confrontations and violence occur almost daily in the Palestinian territories, resulting in injuries and death to children, youth, and adults. In a study of 2481 youths living in the West Bank (WB) and East Jerusalem, EPV was associated with elevated symptoms of general distress (45.7%) and depression (55.2%), and over one-third of the youth had elevated symptoms of anxiety [17]. Dubow et al. [8] investigated the association between exposure to different forms of violence in the social environment among 600 Palestinian youth, and found that EPV was a significant predictor for developing post-traumatic stress symptoms, even after controlling for demographic factors and other forms of violence.

As a whole, most published studies in the area of violence experienced by Palestinians have sampled children and adolescents and focused their inquiry understanding the various psychological sequelae to injuries, including PTSD [18]. Yet, systematic evidence is scarce, which hampers the ability to design and implement interventions that mitigate the psychological consequences of PV for this population. Reliable estimates of the prevalence of PTSD and the identification of risk factors associated with PTSD among children and adolescents exposed to ongoing conflict are needed to inform public policy and to develop context-related and adapted mental health services, trainings for professionals, and research planning [19]. Therefore, we aimed to systematically review the literature to explore the prevalence of PTSD in children and adolescents exposed to PV in the oPt, and identify concurrent factors that might be associated with the development of PTSD in this population.

## Methods

### Search strategy

We followed the Preferred Reporting Items for Systematic Reviews and Meta-Analyses (PRISMA) guidelines [20]. Population-based, observational studies reporting the prevalence of PTSD in Palestinian children and adolescents were identified by two independent researchers following an electronic search from inception until June 2020 in the following databases: PubMed, Embase, PsycInfo, Google Scholar and Cochrane Library. The search terms “stress disorder” and “post-traumatic stress disorder” were combined with “Palestine” or “West Bank” or “Gaza Strip”. Thus, six search queries were conducted in each database (S2 Table). In Google Scholar, findings were ordered according to their relevance and the first 200 hits were screened. The references list of each included article was also reviewed for potentially eligible studies. Peer-reviewed and grey literature were included, if they fulfilled the following criteria: a) studies that aimed to assess PTSD as a result of exposure to political violence (i.e. war, confrontations with the army), b) participants were less than 19 years old at the time of the PTSD assessment, c) PTSD diagnostic tools based on the fourth and fifth editions of the Diagnostic and Statistical Manual of Mental Disorders (DSM-IV and DSM-V). For five of the six studies that had missing information, the data were obtained through contacting the authors, only one author did not reply.

#### Risk of bias assessment

An adjusted version of the Joanna Briggs Institute Prevalence Critical Appraisal Tool was used to assess the risk of bias in the included studies. This tool consists of 10 items that assess the validity and quality indicators specific to studies that report prevalence data (S1 Table) [21]. Item ten (‘Were sub-populations identified using objective criteria?’) was not considered relevant for this review. Ultimately, nine items were rated as yes, no, or unclear. The sum of the “yes” items given to each study was calculated. Item three (‘Was the sample size adequate?’) was rated according to the sample size needed to estimate the prevalence with a precision of 95%, which was at least 369 participants [22].

Both screening and quality assessment were performed by two authors (NA and SP) and discrepancies were resolved by consultation with the other review authors.

We assessed possible publication bias using a scatter plot with the prevalence of each study on the x-axis and the corresponding sample size on the y-axis. Due to missing information on the variance of most prevalence measures, we utilized the studies’ sample sizes instead [23].

#### Descriptive and quantitative analysis

A pooled estimate assuming random effects weighted by sample size was calculated according to the Schmidt-Hunter method [24], and visualized using a forest plot. The random-effects-model was used considering source of variance other than sampling error only, assuming no common effect size [25]. Inter-study heterogeneity was quantified by the *I*^*2*^ value, where *I*^*2*^ values above 75% would suggest high heterogeneity [26]. To further explore sources of heterogeneity, we conducted meta-regression by setting up a linear regression including 25 samples from 21 included studies [27–47]. Prevalence estimates were the dependent variable and all other variables of the subgroup analysis were the independent variables except for gender which we could not include due to limited amount of data. From this model, we calculated the *R2* index in order to quantify the proportion of variance explained by the included covariates.

Subgroup analyses were performed to estimate the pooled prevalence of PTSD with regard to geographic region (WB, GS), survey setting, time elapsed since the exposure to trauma and the assessment of PTSD (during the first six months after the trauma exposure, or six months following the trauma exposure). Studies were classified as being low risk of bias or high risk of bias (quality assessment score ≥7, and ≤6 respectively), representativeness of the sample, survey method (interview, questionnaire), gender, and mean age which was divided into two groups, ≥13, <13 years.

Analyses was performed with Stata/IC 15.1 for Windows (StataCorp LLC, 4905 Lakeway Drive, College Station, TX 77845, USA).

## Results

### Literature search and study characteristics

**Fig 1:**
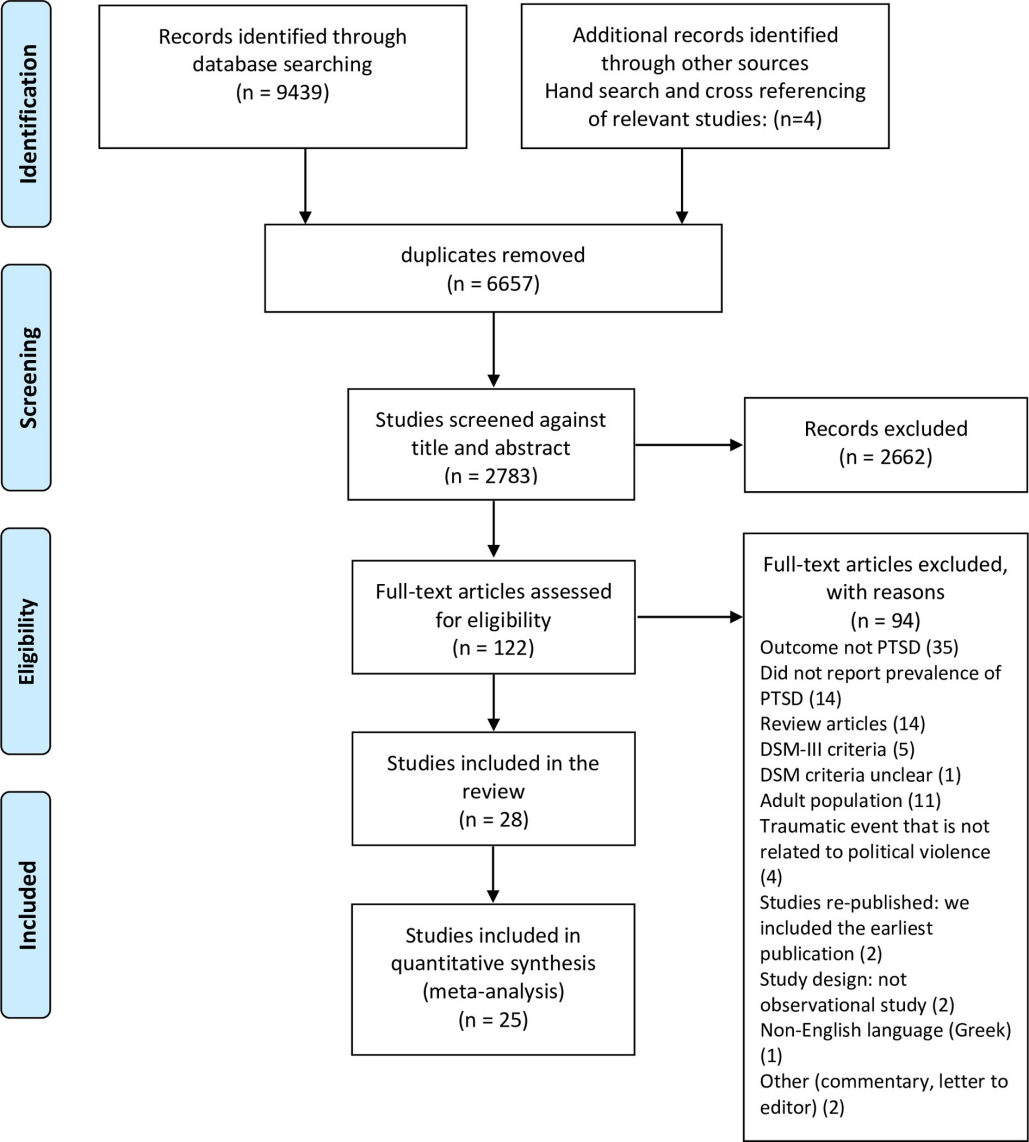
PRISMA flow-chart of the search strategy and study selection.

Fig 1 represents the search and selection process. The search strategy retrieved 9443 studies, and after removing 6657 duplicates, 2786 studies were screened by title and abstract. 2664 studies that did not fulfill our inclusion criteria were excluded and the remaining 122 studies were eligible for full text assessment, of which 28 studies were finally included in this review. Study characteristics are presented in Table 1. The final pool of studies comprised 32 samples from WB and GS and included a total of 15,121 participants (ranging from 54 to 1850 per study). GS was the data collection site for most of the samples (n = 24, 77.4%), while eight samples were from WB (26%).

**Table 1:** Characteristics of the included studies.

Author, year	Region	Setting	Sample size	Response rate [%]	Male [%]	Mean age [SD] [years]	PTSD assessment tool	Assessment method	Time of Data collection	Symptoms duration	Exposure to trauma	Informant	Cut-off score	PTSD [%]	Quality score Out of 9
Abdeen, 2008	WB GS	Schools	1354 746	N/R	55	15.9 (1.0)	UCLA-PTSD-I	Self-report	2004–05	During the last 4 weeks	N/R	Study subjects	N/R	36 35	7
Al-Ghalayini, 2017	GS	Kinder-gartens	420	95	50.4	4.5 (0.7)	CPSS	Interview	May 2015	N/R	1 year prior to survey	Mothers	N/R	6	7
Al-Sheikh, 2017	GS	Schools	400	N/R	50	15.5 (1.7)	PTSD checklist–Arabic version	Self-report	August 2016	Previous month	2014 war	Study subjects	N/R	9.3	7
Elbedour, 2007	GS	Schools	300	76.3	52.8	17.1 (1.5)	PTSD-I	Self-report	2002	1 month after the event and one month prior to survey	2000–02	Study subjects	N/R	68.9^a^	7
El-Khodary, 2019	GS	Schools	1131	91	48.2	13.7 (1.3)	PTSDSS- DSM5	Interview	2012	N/R	War 2012	Study subjects	N/R	53.4	8
Fasfous, 2013	WB	Schools	381	N/R	49	13.0	CPTSD-RI	Self-report	2010	N/R	Lifetime exposure	Study subjects	>40	20.5	6
Khamis, 2008	WB GS	Homes	179	N/R	100%	16.3 (1.6)	DSM-IV	Interview 1 hour	During the 2^nd^ intifada	N/R	1–27 months (M:17.8; SD:7.5)	Study subjects	N/R	76.5 full PTSD	5
Khamis, 2012	GS	Schools	300	N/R	50	12.8 (0.8)	DSM-IV	Interview	2010	N/R	Lifetime exposure	Study subjects	N/R	33	5
Khamis, 2015	GS	Homes	220	93	48.3	13.5	As above	Interview	2013	N/R	War 2012	Study subjects	N/R	19	6
Lavi, 2005^b^	WB	Homes	245	N/R	44	13.5 (0.7)	CPTS-RI	Self-report	Summer 2001	N/R	N/R	Study subjects	60–80	37.1 (very) severe	4
Manzanero, 2017	GS	Community	1,865	99	52	*M*:9.0 (2.0), *F*: 9.2 (2.1)	HTQ-Iraqi version	Self-report	January-May 2015	N/R	Over 6 months after 2014 war	Parents, Tutors	>2.5	27.4^c^	7
Nada, 2010	GS	Schools	368	98	49.2	17.3	DTS	Self-report	2006–07	N/R	6 months prior to survey	Study subjects	N/R	17.1 probable PTSD	7
Pat-Horenczyk, 2009^d^	WB	Schools	1,235	N/R	45.3	15.9 (1.0)	UCLA-PTSD-RI	Self-report	Spring 2004	N/R	N/R	Study subjects	N/R	37.2	5
Qeshta, 2019	GS	Schools	408	97	50	17 (0.8)	PTSD Scale for DSM-IV-Arabic version	Self-report	March 2015	N/R	2014 war	Study subjects	38	16.4	9
Shehadeh, 2015^e^	WB	Homes	79	N/R	55.8	7.7 (1.7)	UCLA-PTSD-RI	Self-report	2012	N/R	N/R	Mothers	>30	25.30	5
Shehadeh, 2016 ^f^	WB	As above	204	N/R	57.4	13.4 (1.94)	UCLA-PTSD-RI	Self-report	2012	N/R	N/R	Study subjects	>38	69.6	5
Thabet, 2002^g^	GS	Community	91	100%	47.0	14.3	CPTSD-RI	Self-report	January- February 2001	N/R	N/R	Study subjects	>40 (very) severe	59	6
Thabet, 2006(a)^h^	GS	Community	420	97	50.1	13.0 (2.5)	CRIES-13	Self-report	July-August 2006	During the past week	Over the last year	Study subjects	>30	65.5	7
Thabet, 2008	GS	Community	197	N/R	N/R	*M*:*12*.*8(2*.*5)*, *F*: *13*.*2(2*.*51)*	CRIES-13	Self-report	June 2006	over the previous 6 months	Over the previous 6 months	Study subjects	>30	70.1^i^	5
Thabet, 2009	GS	Schools	412	100%	48.5	13.7 (1.1)	SCID	Interview	During Al Aqsa Intifada	N/R	Preceding 24 months	Study subjects	N/R	30.8^J^	8
Thabet, 2011	GS	Homes	410	100%	54.6	12.9 (3.9)	UCLA PTSD	Self-report	May-June 2009	N/R	6 months prior to survey	Study subjects	N/R	9.8 full PTSD	8
Thabet, 2014	GS	Schools	358	100%	44.1	16.7 (0.8)	UCLA PTSD	Self-report	April 2009	N/R	3 months after 2008–9 war	Study subjects	N/R	29.8^k^ full PTSD	7
Thabet, 2015(a)^L^	GS	Community	502	N/R	50	12.6 (2.2)	UCLA PTSD	Self-report	January 2013	N/R	November 2012 war	Study subjects	N/R	36 full PTSD	6
Thabet, 2015(b)	GS	Community	462	97	51.9	7–18	UCLA PTSD	Self-report	2010	N/R	16 months prior to survey- 2008 war	Study subjects	N/R	12.4 full PTSD	7
Thabet, 2016	GS	Summer-camps	251	97	51.4	11.2 (2.7)	CRIES-8	Self-report	Summer 2011	N/R	Previous week-related to the 2009 war	Study subjects	>17 likelihood of PTSD	59	5
**Grey literature**															
Altawil, 2008	GS	Schools	1137	N/R	43.8	14.4 (1.78)	SPTSDS	Self-report	2006–07	N/R	2000–05	Study subjects	N/R	41	5
Qouta, 2004	GS	Schools	944	N/R	49.7	15.1 (1.5)	CPTS-RI	Self-report for older children, Interview for younger children	N/R	N/R	lifetime trauma exposure	Study subjects	>40 severe	32.7^m^	5
Thabet, 2006(b)	WB GS	Schools	150 200	100%	51.7 51.3	10 (1.6) 9.5 (2.2)	IES	Self-report	Feb-March, 2003	N/R	N/R	Study subjects	>40	34 39	3

Abbreviations: SD, Standard Deviation; N/R, Not Reported; UCLA-PTSD-I, University of California at Los Angeles Posttraumatic Stress Disorder Index for DSM-5; DTS, The Davidson Trauma Scale; CPSS, Child PTSD Symptom; SPTSDS, Symptoms PTSD Scale; CPTSD-RI, Children Post Traumatic Stress Reaction Index; HTQ, Harvard Trauma Questionnaire; UCLA PTSD RI, Reaction Index; SCID, Structured Clinical Interview; IES, impact of Event Scale; CRIES, The children’s revised impact of events scale.

^a^males 68.9%, females 69.0%,

^b^study included comparison sample of Palestinians in Israel (n = 300),

^c^males 28.3% females 26.5%,

^d^comparison with Israeli sample (n = 1016),

^e^study included comparison group (n = 89),

^f^Study included comparison group (n = 110),

^g^study included control group (n = 89),

^h^males 61.9% females 70.1%,

^i^males 69%, females 71.1%,

^J^males 30%, females 31.6%,

^k^males 26% females 33%, Study design survey, except for ^d^escriptive study,

^L^descriptive analytical study,

^m^males 42.1% females 57.9%.

Three studies included comparison samples from other regions as follows: Palestinians living in Israel [48], an Israeli-Jewish sample [38], and a sample from South-Lebanon [35]. Three studies used comparison samples of unexposed subjects from the respective region [40, 46, 49]. The mean age of the included samples ranged from 4.5 years to 17.1 years. Most studies included both genders, except for one study which exclusively enrolled boys [34]. One study did not report the gender split [50]. Data were collected mostly from the children and adolescents themselves (n = 25, 89%), two studies collected data from the mothers, and in one study the informants were parents and tutors. Fifty percent of the studies were conducted in schools, six studies (21.4%) in the community, and another six studies at the home of the participants. Most of the studies (n = 20, 71.5%) were conducted more than six months following the exposure to trauma, and eight (28.5%) studies were conducted within the first six months of exposure.

All PTSD assessment tools correspond to the DSM-IV criteria except for one study that used the DSM-V criteria [31]. Different cut-off scores for identification of PTSD were used for the same tool in each study. Only seven (25%) studies used a PTSD assessment tool that was validated in the Palestinian population.

### Risk of bias assessment

The results of the risk of bias assessment are presented in the (S1 Table). Thirteen studies (46%) scored seven or higher out of nine, and 17 studies (60%) had an adequate sample size. Thirteen studies (46%) had a representative sample. The scatter plot did not provide evidence for publication bias (see S1 Fig).

#### Prevalence of PTSD

**Fig 2:**
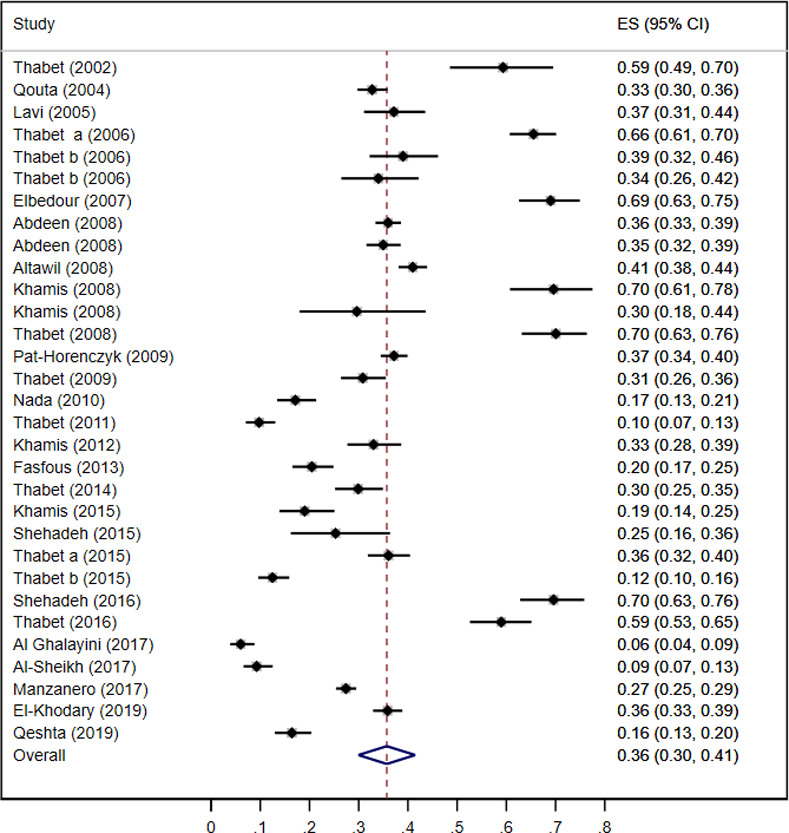
Forest plot of the estimated point prevalence of PTSD of the included studies. Abbreviations: ES, Estimate for the Prevalence; CI, Confidence Interval.

Overall, the reported prevalence of PTSD ranged from 6% to 70%. The estimated point prevalence combining all studies was 36% (95% CI 30–41%; *I*^*2*^ 98.6%) (see Fig 2). Similar results were generated for GS 36% (95% CI 29–43%; *I*^*2*^ 98.8%) and WB 36% (95% CI 28–45%; *I*^*2*^ 96%). When including only studies with a representative sample (as rated in the quality assessment), the prevalence for GS decreased to 24%, and for WB there was one study only in this category reporting a prevalence of 36%.

Table 2 presents the results for the sub-group analysis. The estimates according to region and method of PTSD assessment did not differ. Pooled estimates were highest for studies conducted at schools (*p*<0.001), and studies conducted within the first 6 months of exposure to trauma (*p*<0.001). PTSD prevalence was lower among studies using a representative sample (*p*<0.001), and among those with low risk of bias (*p*<0.001). There were slight differences in PTSD estimates between sexes (*p* = 0.001), and between the age groups (*p*<0.001). Yet, relatively wide confidence intervals were obtained for most of the sub-groups.

**Table 2:** Results of sub-group analysis according to potential factors associated with PTSD in children and adolescents exposed to PV in Palestine.

	K*	Sample size	Prevalence %	95% CI	*P* value
**Region**					
WB	8	3702	36	28–45	1
GS	23	11419	36	29–43	
**PTSD assessment**					
Self-report	20	9495	37	29–44	1
Interview	8	2753	37	21–52	
**Survey setting**					
Home /Community	12	4410	43	33–55	<0.001
School	17	10061	31	25–37	
**Time from exposure**					
6 months	8	3060	50	39–62	<0.001
>6 months	23	12061	31	25–37	
**Sample representative**					
Yes	16	9421	25	19–31	<0.001
No	15	5700	48	38–57	
**Risk of bias**					
Low	14	8821	28	20–37	<0.001
High	17	6300	42	35–48	
**Gender**					
Female	6	2038	48	32–64	0.001
Male	6	2234	43	29–56	
**Mean age**					
<13 years	8	4062	37	31–44	<0.001
≥13 years	21	10412	30	19–41	

Abbreviations: PV, Political Violence; WB, West Bank; GS, Gaza Strip.

*Number of samples.

Results of the meta-regression showed a relatively high share of explained variation with an *R*^*2*^ of 56.6%, while the only significant variable was the representativeness of the sample (*p* = 0.011). This also had the highest impact on the estimated prevalence (S3 Table).

#### Exposure to political violence and PTSD

The Gaza Traumatic Events Checklist (GTEC) was the most commonly used tool to assess the EPV (n = 14) [28–30, 33, 37, 39, 41–45, 47, 50, 51]. Modifications were made to the number and type of items examined in the different studies. Most of the studies reported a significant positive relationship between EPV and the development of PTSD. One study reported no significant relationship between proximity to the traumatic event and PTSD [34]. Fourteen studies reported that the most prevalent types of traumatic events were an indirect exposure, such as hearing drones and shelling artillery, and exposure to distressful events through the media [28, 29, 31, 33, 37, 39, 41–45, 47, 51, 52]. Similar results were found among children and adolescents witnessing their fathers being detained by Israeli soldiers (n = 2) [40, 49].

#### Socio-demographic factors associated with EPV

*Gender*. Boys reported higher EPV than girls across 13 studies [27–29, 33, 36–39, 43, 44, 47, 50, 52], while four studies reported no differences in exposure across gender [42, 45, 51, 53].

*Place of residence*. Participants from WB reported higher exposure than their counterparts in GS [27, 41]. In GS, participants living in villages reported higher exposure than those living in refugee camps and cities [37, 47]. However, two studies reported contradicting results [43, 44], and one study [51] found no difference.

*Economic status*. Three studies found that family income was negatively associated with higher trauma exposure [28, 37, 42]. However, one study reported no significant association [44].

*Age*. Six studies investigated differences in exposure according to age groups: no differences were found in three of these studies [44, 51, 52]. However, a higher age group was associated with higher exposure to traumatic events [29, 43], and one study reported that younger children were more frequently exposed than older ones (9–12 compared with 13 years) [42].

#### Socio-demographic factors associated with PTSD

*Gender*. Seven studies reported proportions for PTSD according to gender [32, 42, 43, 47, 50, 53, 54] and 14 studies stated that there were no significant associations between both [27, 32, 33, 36, 37, 39, 40, 42, 43, 45, 49, 50, 52, 53]. In contrast, two studies reported significantly higher prevalence among boys [28, 29] and four studies reported higher prevalence among girls [38, 44, 47, 54].

*Age*. Eight studies reported no association between age and PTSD [34, 39, 40, 43, 44, 50, 52, 53]. One study reported PTSD was higher in children aged 9–12 than children in the age group ≥13 years [42]. Another study found that children aged 7–11 years had higher PTSD prevalence compared with older age groups [51]. PTSD was significantly associated with older age children (5–6 years old compared with 3–4 years old) [28], and with adolescents (16 years) compared with younger age of 13–15 years [29].

*Economic status and family size*. Three studies reported low-income of families was positively associated with PTSD [39, 44, 51], and another three studies found no significant relationship [28, 34, 43]. Adolescents reporting high levels of economic pressure (difficulties to pay bills or to meet daily family needs) were at higher risk for developing PTSD [35]. Having more than eight siblings was also associated with PTSD among children living in GS by one study [28]. In contrast, one study found a similar association in children living with less than four siblings [42], and one study no association [43]. One study found that living with extended family increased the risk for developing PTSD among teenagers [40].

*Place of residence*. Three studies included samples from both WB and GS and reported no differences in PTSD prevalence [27, 34, 41]. In WB, children living in rural areas had higher rates of PTSD as compared to those living in refugee camps [49]. Adolescents from villages reported higher prevalence of PTSD than those from cities [40]. In GS on the other hand, children living in the city had higher PTSD prevalence than those living in village or refugee camps [44, 51]. No significant difference was found between three types of residence (city/village/refugee camp) in one study [43]. More parental support was associated lower PTSD in adolescents in GS [43]. One study found a positive correlation between PTSD and social support and family support [29]. Adolescents who believed more in fate and used more negative coping strategies were more likely to be diagnosed with PTSD [34]. However, religiosity and ideology were not associated with PTSD in one study [35].

*Comorbidities with PTSD*. Comorbidity between PTSD and anxiety and depression at the same time was found in 3 studies [32, 34, 35], and two studies reported the comorbidity between PTSD and anxiety only [39, 42]. Neuroticism [36], and somatic complaints [27, 38] were found to co-occur with PTSD, as well as attention-deficit hyperactivity disorder [45].

*Coping strategies*. PTSD was positively associated with positive appraisal coping [53], and with emotion-focused coping strategies, while problem-focused strategies were negatively associated with PTSD in children [36].

## Discussion

We identified 28 eligible studies on children and adolescents in Palestine (n = 15,121) with an overall pooled PTSD prevalence of 36%. This is lower than the 47% reported in a 2009 meta-analysis of 7,920 children exposed to armed conflicts worldwide [55], and almost twice as much as the reported PTSD prevalence in a meta-analysis of studies in trauma-exposed children and adolescents (n = 3563, 15.9%). Yet this meta-analysis included studies applying well-established interviews, and children exposed to war were underrepresented [56]. A recent study conducted in war-torn Syria among 1369 students (mean age = 16), reported a higher PTSD prevalence of 53% [57]. The wide range of PTSD observed in our review (6%-70%) is consistent with a 2012 systematic review of mental health of children and adolescents in areas affected by armed conflicts in the Middle-East (23–70%) [58]. However, we were able to show that half of the variability (*R*^*2*^ = 56.6%) can be exclaimed by factors including sample representativeness, the study design and the time between exposure and PTSD assessment.

Our sub-group analysis suggested evidence of between-group differences in PTSD prevalence across several variables (e.g. age, survey setting, time between survey and exposure, risk of bias).

A closer analysis, though, suggests that a better approach is to consider the interactive relationships among some of these factors. Visual inspection of the included studies revealed large discrepancies in assessing and reporting the association between different risk factors and the development of PTSD. For example, the majority of the studies reported a significant contribution of scale and type of war-related traumatic events to the severity of PTSD symptoms, indicating the overwhelming experience of the trauma. This is also consistent with the dose-effect relationship between repeated exposure to trauma and the development of mental health adversities [13, 59, 60]. However, while most of the studies reported that boys had higher EPV, adding participant gender to the sub-group analysis showed that girls were at higher risk for developing PTSD than boys. One explanation for this apparent inconsistency the tendencies for boys to externalize their behaviors following trauma rather internalizing them [61], hence accounting for inconsistencies between exposure rates across gender and PTSD prevalence. Additionally, neurobiological biomarkers and the developmental age are suggested to explain some of the gender differences [62]. Similar to other risk factors explored in this review, small number of studies explored the relationship between gender and EPV and between PTSD, providing equivocal findings (see results section). Further research is needed to understand the reasons for these discrepancies in the Palestinian context. The sub-group analysis also demonstrated a decline in PTSD prevalence after six months of exposure to trauma. Despite the decline, the prevalence remained relatively high, suggesting that without proper interventions it is unlikely that PTSD symptoms would quickly remit. We were not able to control for additional factors. Most socio-economic factors were investigated by small number of studies, while providing different assessment measures and results for most of these risk factors have not always been consistent, which did not allow further analysis to determine effect sizes.

Besides understanding patterns of the EPV and the mental health consequences, the Palestinian context brings unique challenges due to the duration and pervasiveness of the EPV within the oPt [63], and in essence, is characterized by chronic and multi-generational trauma [63]. The ongoing nature of the conflict means recency and accumulative effects of traumatic exposure are thus associated with increased prevalence of PTSD [64, 65], which reflects the problematic definition of PTSD as it assumes the “post” conflict conditions. This situation not only increases the risk of developing PTSD but also hinders the ability to recover among youth who experience additional traumatic events after the trauma that triggered the onset of PTSD [66]. This emphasizes the need to investigate the health consequences of the collective experience and not only focus on the individual response to exposure to trauma. The cumulative effect of multiple types of trauma was investigated in few of the included studies, see for example El-Khodary and Samara [31].

An additional aspect pertaining to the contextual frame of measuring PTSD is its historical conceptualization in Western psychiatry. As such, the wide use of these concepts including PTSD in non-Western populations endorse the Western ontology and value system in these populations [67]. This also applies to the PTSD assessment tools used in Palestine. We found a small number of studies that utilized assessment tools that were validated in the Palestinian society and mostly are based on self-report rather than clinical interviews, which may result in inadequacies in diagnosing PTSD in this population [68].

Importantly, we are not arguing for the exclusion of PTSD analyses within the oPt. Instead, we are making the case for reconsidering and re-evaluating the methods and assessment process being utilized therein, and to devote resources toward the development of brief screening tools specifically adapted for use in the wake of acute events (such as assaults on Gaza), and that capture contextually-specific aspects of resilience. A comprehensive and systematic investigation of the social, political, cultural and economic factors is necessary to promote intervention programs as well as prevention measures.

Finally, this systematic review may provide a framework for future PTSD research on children and adolescents in the oPt. Following changes in the diagnostic criteria for PTSD in DSM-V, and in the 11^th^ edition of the International Classification of Diseases (ICD-11) [69], estimates among children and adolescents may differ significantly based on the use of different diagnostic systems, which could affect both the research strategy and the provision of treatment and therapy [70].

### Strengths and limitations of this review

To our knowledge, this is the first systematic literature review and meta-analysis of PTSD in Palestinian children and adolescents exposed to PV. However, limitations of this study should be considered when interpreting the results. The high heterogeneity between the included studies partly determines the difficulty in drawing overall conclusions [26]. *I²* reflects what proportion of the variation in observed effects is due to variation in true effects [71]. Therefore, the actual interpretation of an *I²* value is not straightforward [72]. Assessment of a publication bias could not be done with a standard Funnel plot due to missing information on the variance. Instead, we used a plot presenting the study precision by the sample size. All included studies applied a cross-sectional design, thus limiting their representativeness, and the percentage of children and adolescents recovering from PTSD remain unclear. Additionally, most of the studies presented the PTSD prevalence as a point estimate, yet, none of the included studies provided an estimate of uncertainty like confidence intervals. When the overall PTSD prevalence was not available, we included the highest cut-off score for PTSD diagnosis in the analysis, thus, not including other sub-groups who were also reported to have PTSD at lower cut-off scores (i.e. moderate PTSD). Three of the included studies reported the prevalence for a comparison sample. In this review, we reported the prevalence of the main sample only.

## Conclusions

Notwithstanding the limitations, the PTSD prevalence of 36% reported in this systematic review indicates the high relevance of trauma and the high burden for mental health in this population. The findings also highlight the need for developmentally appropriate, and locally adapted assessment tools for psychological adversities in the Palestinian context. A further prospective investigation is warranted to understand the pre-trauma and post-trauma risk factors in this community not only to develop a better interventional and prevention programs, but also to help identifying particularly vulnerable groups within the Palestinian society who might be at higher risk to develop psychological pathology following exposure to trauma, and to obtain appropriate treatment.

## Supporting information

S1 Table Risk of bias assessment of the included studies. (PDF)

S2 Table Search strategy. (PDF)

S3 Table Results of meta-regression of 25 samples of the included studies. (PDF)

S1 Checklist PRISMA 2009 checklist. (PDF)

S1 Data Study protocol. (PDF)

S1 Fig Scatter plot of publication bias. (TIF)
