# Supplementary material for: Prevalence of post-traumatic stress disorder among Palestinian children and adolescents exposed to political violence: A systematic review and meta-analysis
Source: PLoS One. 2021 Aug 26;16(8):e0256426. doi: 10.1371/journal.pone.0256426 (PMC8389374; doi:10.1371/journal.pone.0256426)
Supplement: S1 Table — (PDF) [file pone.0256426.s001.pdf]

S1 Table. Risk of bias assessment of the included studies

Prevalence of post-traumatic stress disorder among Palestinian children and adolescents exposed to political violence: A systematic review

Study	1- Was the sample representative of the target population?	2- Were study participants recruited in an appropriate way?	3- Was the sample size adequate?	4- Were the study subjects and the setting described in detail?	5- Was the data analysis conducted with sufficient coverage of the identified sample?	6- Were objective, standard criteria used for the measurement of the condition?	7- Was the condition measured reliably?	8- Was there appropriate statistical analysis?	9- Are all important confounding factors/subgroups/ differences identified and accounted for?
**Abdeen 2008**	Yes	yes	yes	Yes	no	yes	yes	yes	unclear
**AlGhalayini 2017**	yes	yes	yes	yes	yes	yes	unclear	yes	Unclear
**Qeshta 2019**	yes	yes	yes	yes	yes	yes	yes	yes	yes
**Al-Sheikh 2017**	yes	yes	yes	Yes	no	yes	yes	yes	unclear
**Altawil 2008**	yes	Unclear	yes	unclear	no	yes	yes	yes	no
**Elbedour 2007**	no	yes	no	yes	yes	yes	yes	yes	yes
**El-Khodary 2019**	yes	yes	yes	yes	yes	yes	yes	yes	unclear
**Fasfous 2013**	no	yes	yes	yes	no	yes	yes	yes	unclear
**Khamis 2012**	No	yes	no	yes	no	yes	yes	yes	unclear
**Khamis 2015**	yes	yes	no	yes	yes	yes	yes	no	unclear
**Khamis 2008**	no	yes	no	yes	no	yes	yes	yes	unclear
**Lavi 2005**	no	Unclear	no	yes	no	yes	yes	yes	unclear
**Manzanero 2017**	no	unclear	yes	yes	yes	yes	yes	yes	yes
**Nada 2010**	yes	yes	yes	yes	yes	yes	unclear	yes	unclear
**Pat-Horenczyk 2009**	no	no	yes	yes	no	yes	yes	yes	unclear
**Qouta 2004**	unclear	Unclear	Yes	Yes	no	Yes	Yes	Unclear	Yes
**Shehadeh 2015**	unclear	yes	no	yes	unclear	yes	yes	yes	unclear
**Shehadeh 2016**	unclear	yes	no	yes	unclear	yes	yes	yes	unclear
**Thabet 2006(a)**	unclear	no	yes	yes	yes	yes	yes	yes	yes
**Thabet 2002**	unclear	yes	no	yes	yes	yes	yes	yes	no
**Thabet 2008**	no	yes	no	unclear	no	yes	yes	yes	yes
**Thabet 2009**	yes	unclear	yes	yes	yes	yes	yes	yes	yes
**Thabet 2015(a)**	yes	unclear	yes	yes	no	yes	yes	yes	unclear
**Thabet 2015(b)**	yes	unclear	yes	yes	yes	yes	yes	yes	no
**Thabet 2011**	yes	yes	yes	yes	yes	yes	yes	yes	no
**Thabet 2014**	yes	unclear	no	yes	yes	yes	yes	yes	yes
**Thabet 2016**	no	no	no	yes	yes	yes	yes	yes	unclear
**Thabet 2006(b)**	unclear	unclear	no	yes	yes	unclear	unclear	yes	unclear
