# Supplementary material for: Prevalence of post-traumatic stress disorder among Palestinian children and adolescents exposed to political violence: A systematic review and meta-analysis
Source: PLoS One. 2021 Aug 26;16(8):e0256426. doi: 10.1371/journal.pone.0256426 (PMC8389374; doi:10.1371/journal.pone.0256426)
Supplement: S2 Table — (PDF) [file pone.0256426.s002.pdf]

S2 Table: Search strategy

Prevalence of Post-Traumatic Stress Disorder among Palestinian children and adolescents exposed to political violence: A Systematic Review

Database	Search terms	Search details	Additional criteria
**Pubmed**	stress disorders, palestine	("stress disorders, traumatic"[MeSH Terms] OR ("stress"[All Fields] AND "disorders"[All Fields] AND "traumatic"[All Fields]) OR "traumatic stress disorders"[All Fields] OR ("stress"[All Fields] AND "disorders"[All Fields]) OR "stress disorders"[All Fields]) AND palestine[All Fields]	No restrictions
	Post-traumatic stress disorder, palestine	("stress disorders, post-traumatic"[MeSH Terms] OR ("stress"[All Fields] AND "disorders"[All Fields] AND "post-traumatic"[All Fields]) OR "post-traumatic stress disorders"[All Fields] OR ("post"[All Fields] AND "traumatic"[All Fields] AND "stress"[All Fields] AND "disorder"[All Fields]) OR "post traumatic stress disorder"[All Fields]) AND palestine[All Fields]	No restrictions
	Stress disorders, west bank	("stress disorders, traumatic"[MeSH Terms] OR ("stress"[All Fields] AND "disorders"[All Fields] AND "traumatic"[All Fields]) OR "traumatic stress disorders"[All Fields] OR ("stress"[All Fields] AND "disorders"[All Fields]) OR "stress disorders"[All Fields]) AND ("middle east"[MeSH Terms] OR ("middle"[All Fields] AND "east"[All Fields]) OR "middle east"[All Fields] OR ("west"[All Fields] AND "bank"[All Fields]) OR "west bank"[All Fields])	No restrictions
	post-traumatic stress disorder, West Bank	("stress disorders, post-traumatic"[MeSH Terms] OR ("stress"[All Fields] AND "disorders"[All Fields] AND "post-traumatic"[All Fields]) OR "post-traumatic stress disorders"[All Fields] OR ("post"[All Fields] AND "traumatic"[All Fields] AND "stress"[All Fields] AND "disorder"[All Fields]) OR "post traumatic stress disorder"[All Fields]) AND ("middle east"[MeSH Terms] OR ("middle"[All Fields] AND "east"[All Fields]) OR "middle east"[All Fields] OR ("west"[All Fields] AND "bank"[All Fields]) OR "west bank"[All Fields])	No restrictions
	Stress disoreders, gaza strip	("stress disorders, traumatic"[MeSH Terms] OR ("stress"[All Fields] AND "disorders"[All Fields] AND "traumatic"[All Fields]) OR "traumatic stress disorders"[All Fields] OR ("stress"[All Fields] AND "disorders"[All Fields]) OR "stress disorders"[All Fields]) AND ("middle east"[MeSH Terms] OR ("middle"[All Fields] AND "east"[All Fields]) OR "middle east"[All Fields] OR ("gaza"[All Fields] AND "strip"[All Fields]) OR "gaza strip"[All Fields])	No restrictions
	post-traumatic stress disorder, gaza strip	("stress disorders, post-traumatic"[MeSH Terms] OR ("stress"[All Fields] AND "disorders"[All Fields] AND "post-traumatic"[All Fields]) OR "post-traumatic stress disorders"[All Fields] OR ("post"[All Fields] AND "traumatic"[All Fields] AND "stress"[All Fields] AND "disorder"[All Fields]) OR "post traumatic stress disorder"[All Fields]) AND ("middle east"[MeSH Terms] OR ("middle"[All Fields] AND "east"[All Fields]) OR "middle	No restrictions

		east"[All Fields] OR ("gaza"[All Fields] AND "strip"[All Fields]) OR "gaza strip"[All Fields])	
**APA psyc NET**<br><br>• PsycINFO<br>• PsycARTICLES<br>• PsycBOOKS<br>• PsycEXTRA<br>• PsycTESTS	stress disorders AND Palestine		No restrictions
	Post-traumatic stress disorder, Palestine		No restrictions
	stress disorders AND West Bank		No restrictions
	Post-traumatic stress disorder AND West Bank		No restrictions
	Stress disorders AND gaza strip		No restrictions
	Post-traumatic stress disorder AND gaza strip		No restrictions
**Embase**	Stress disorder, Palestine		No restrictions
	Posttraumatic stress disorder, Palestine		No restrictions
	Stress disorder, west bank		No restrictions
	Posttraumatic stress disorder, west bank		No restrictions
	Stress disorder, gaza strip		No restrictions
	Posttraumatic stress disorder. Gaza strip		No restrictions
**Google scholar**	stress disorders, palestine		No restrictions
	Post-traumatic stress disorders, Palestine		No restrictions
	stress disorders, west bank		No restrictions
	Post-traumatic stress disorders, west bank		No restrictions
	stress disorders, Gaza strip		No restrictions
	Post-traumatic stress disorders, Gaza strip		No restrictions
