# Supplementary material for: Prevalence of post-traumatic stress disorder among Palestinian children and adolescents exposed to political violence: A systematic review and meta-analysis
Source: PLoS One. 2021 Aug 26;16(8):e0256426. doi: 10.1371/journal.pone.0256426 (PMC8389374; doi:10.1371/journal.pone.0256426)
Supplement: S3 Table — (PDF) [file pone.0256426.s003.pdf]

S3 Table. Results of meta-regression of 25 samples of the included studies

**Variable**		**Coefficient**	***P*-Value**
**Region**	West Bank	-11.60	0.196
	Gaza Strip	Ref	
**Assessment**	Self-report	-2.09	0.761
	Interview	Ref	
**Survey-setting**	Home/ Community	Ref	0.961
	School	-0.36	
**Time from exposure**	6 months	-9.44	0.231
	>6 months	Ref	
**Sample**	Representative	-23.49	0.011
	Not representative	Ref	
**Quality assessment score**	High (≥7)	Ref	
	Low (<7)	4.71	0.56
**Age**	<13 years	Ref	0.192
	≥13 years	9.98	
**Constant**		50.26	0.001
**Number of samples**	**25**		
**R^2**	**56.9%**		

Prevalence estimates were the dependent variable and all other variables of the subgroup analysis were the independent variables except for gender which we could not include due to limited amount of data.
