# Supplementary material for: Prevalence of post-traumatic stress disorder among Palestinian children and adolescents exposed to political violence: A systematic review and meta-analysis
Source: PLoS One. 2021 Aug 26;16(8):e0256426. doi: 10.1371/journal.pone.0256426 (PMC8389374; doi:10.1371/journal.pone.0256426)
Supplement: S1 Data — (PDF) [file pone.0256426.s005.pdf]

S1 Data: Prevalence of Post-Traumatic Stress Disorder (PTSD) among Palestinian children and adolescents exposed to political violence: A Systematic Review protocol

Authors: Nisreen Agbaria, Stephanie Petzold, Andreas Deckert, Nicholas Henschke, Guido Veronese, Peter Dambach, Thomas Jaenisch, Olaf Horstick, Volker Winkler

Corresponding author: Nisreen Agbaria, Email: nigbaria@gmail.com

Abstract

Rationale: Children and adolescents living in war torn areas are exposed to political violence which affect their well-being and various contexts of their lives such as the family, school, peer networks, and the entire environment in which children and youth are raised. The protracted Israel-Palestinian conflict has drawn an increased interest in this situation's negative impact on children's and adolescent's physical and mental health. Most of the published studies in this area have included both children and adolescents, and focused on individual injuries and various psychological sequelae including PTSD.

Research question:

- What is the prevalence of PTSD among Palestinian children and adolescents exposed to political violence?
- What are the factors associated exposure to political violence and with the development of PTSD in this population?

Search strategy:

PubMed, Embase, APA PsycNet, Google Scholar and Cochrane library will be searched from inception using a search strategy designed to capture all the relevant literature that report the prevalence of PTSD in Palestinian children and adolescents exposed to political violence. The search strategy will be designed to access published research in three different stages:

(1) Search of Pubmed to identify relevant keywords in title and abstract
(2) Terms identified in this way will be used in an extensive search of the literature in the above-named databases.
(3) Reference lists of the articles collected from those identified in stage two will be searched as well.

An example of search strategy: Pubmed

Search terms: stress disorders, Palestine

Search details: ("stress disorders, traumatic"[MeSH Terms] OR ("stress"[All Fields] AND "disorders"[All Fields] AND "traumatic"[All Fields]) OR "traumatic stress disorders"[All Fields] OR ("stress"[All Fields] AND "disorders"[All Fields]) OR "stress disorders"[All Fields]) AND palestine[All Fields]

Additional criteria: no restriction

Eligibility criteria:

We will include studies published in peer-reviewed journal and grey literature, and include the following:

Population: participants were exposed to conflict-related trauma, and were less than 19 years old at the time of the PTSD measurement

Study design: population-based, observational studies using the PTSD diagnostic tools based on the fourth and fifth editions of the Diagnostic and Statistical Manual of Mental Disorders (DSM-IV) and (DSM-V). If the same data were reported in multiple manuscripts, the earliest publication was included.

Outcome measures: the article and/or the study author(s) reported the prevalence of PTSD diagnoses. Studies solely performing a psychometric evaluation of the diagnostic interview were excluded.
Publication date: no restrictions Language: no restrictions

Data management and extraction: screening and data extraction will be conducted using an online tool (https://www.covidence.org). Two reviewers will first review the titles of articles yielded by the search, and then the abstracts of articles of potential relevance. The full texts of potentially eligible papers will be assessed, and data will be extracted by two reviewers. Conflicts in selections and quality assessment will be resolved by discussion and consultation with the other coauthors.

Assessment of methodological quality: This will be done using the Joanna Briggs Institute Prevalence Critical Appraisal Tool. This tool was specifically developed to assess the quality and the risk of bias of studies reporting prevalence estimates. Screening and quality assessment of articles will be performed by two reviewers (NA and SP), and in case of conflicts, they will be resolved by consultation with the other coauthors.

Data synthesis: we will conduct a quantitative analysis to calculate the pooled prevalence of PTSD for the total sample, and according to different sub-groups that will be defined during the data extraction. We will provide a descriptive summary of the co-factors associated with PTSD and with the exposure to political violence.

Systematic review registration: This systematic review protocol is not registered.
