# Supplementary figures and images for: Prevalence of post-traumatic stress disorder among Palestinian children and adolescents exposed to political violence: A systematic review and meta-analysis
Source: PLoS One. 2021 Aug 26;16(8):e0256426. doi: 10.1371/journal.pone.0256426 (PMC8389374; doi:10.1371/journal.pone.0256426)

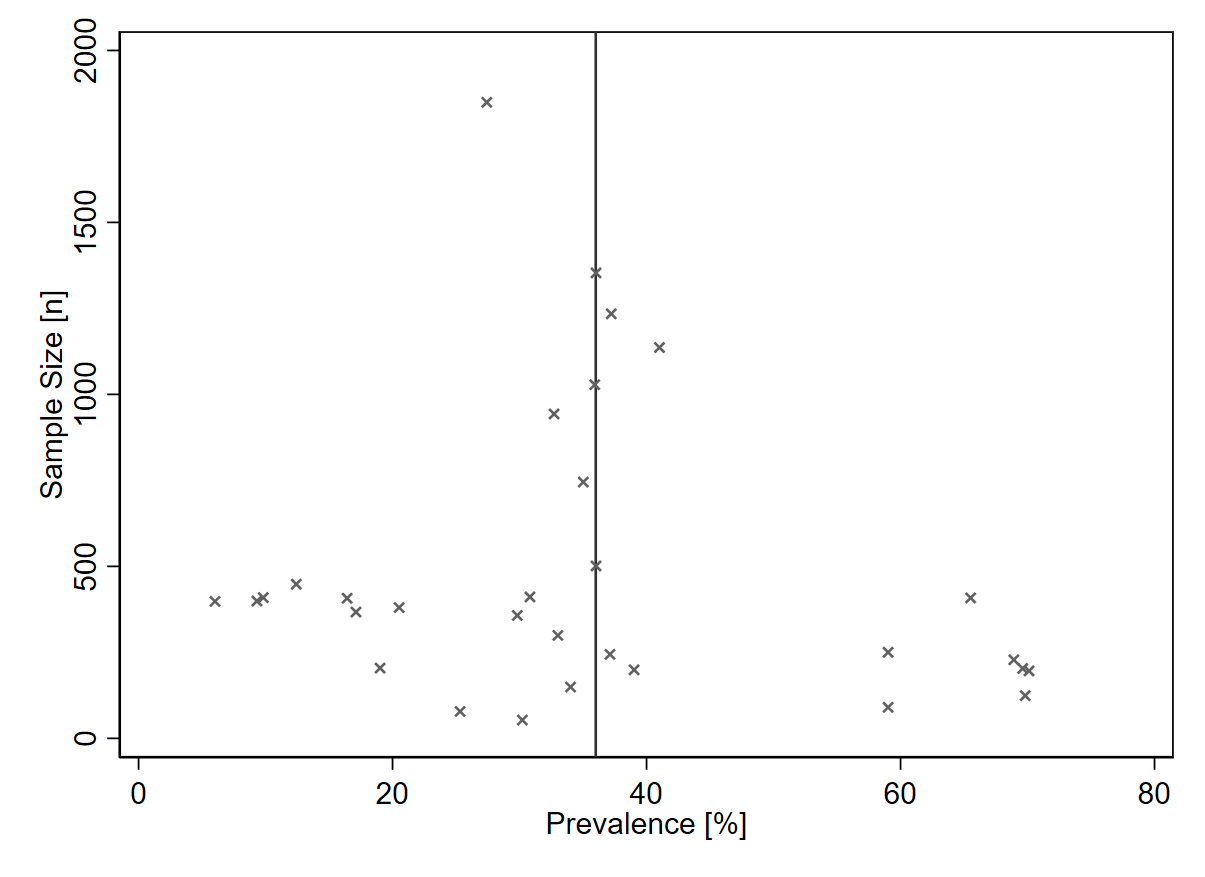

Supplement: S1 Fig — (TIF) [file pone.0256426.s006.tif]
